# The Effect of Side of Implantation on the Cortical Processing of Frequency Changes in Adult Cochlear Implant Users

**DOI:** 10.3389/fnins.2020.00368

**Published:** 2020-04-29

**Authors:** Chun Liang, Lisa H. Wenstrup, Ravi N. Samy, Jing Xiang, Fawen Zhang

**Affiliations:** ^1^Department of Communication Sciences and Disorders, University of Cincinnati, Cincinnati, OH, United States; ^2^Child Psychiatry and Rehabilitation, Affiliated Shenzhen Maternity & Child Healthcare Hospital, Southern Medical University, Shenzhen, China; ^3^Department of Otolaryngology-Head and Neck Surgery, University of Cincinnati, Cincinnati, OH, United States; ^4^Department of Pediatrics, Cincinnati Children’s Hospital Medical Center, Cincinnati, OH, United States

**Keywords:** cochlear implant, frequency change detection, acoustic change complex, standardized low-resolution brain electromagnetic tomography (sLORETA), temporal lobe, frontal lobe

## Abstract

Cochlear implants (CI) are widely used in children and adults to restore hearing function. However, CI outcomes are vary widely. The affected factors have not been well understood. It is well known that the right and left hemispheres play different roles in auditory perception in adult normal hearing listeners. It is unknown how the implantation side may affect the outcomes of CIs. In this study, the effect of the implantation side on how the brain processes frequency changes within a sound was examined in 12 right-handed adult CI users. The outcomes of CIs were assessed with behaviorally measured frequency change detection threshold (FCDT), which has been reported to significantly affect CI speech performance. The brain activation and regions were also examined using acoustic change complex (ACC, a type of cortical potential evoked by acoustic changes within a stimulus), on which the waveform analysis and the standardized low-resolution brain electromagnetic tomography (sLORETA) were performed. CI users showed activation in the temporal lobe and non-temporal areas, such as the frontal lobe. Right-ear CIs could more efficiently activate the contralateral hemisphere compared to left-ear CIs. For right-ear CIs, the increased activation in the contralateral temporal lobe together with the decreased activation in the contralateral frontal lobe was correlated with good performance of frequency change detection (lower FCDTs). Such a trend was not found in left-ear CIs. These results suggest that the implantation side may significantly affect neuroplasticity patterns in adults.

## Introduction

Cochlear implants (CIs) have been successful in providing auditory sensation to individuals with severe to profound hearing loss. Recently, more and more CIs have been used in both children and adults with hearing loss. However, there is large variability in CI users’ speech outcomes. Previous studies have suggested that CI outcomes could be affected by many factors such as the duration of deafness, age at implantation, the duration of CI use, cognitive ability, and electrode placement (Finley and Skinner, 2008; Lazar et al., 2010; Lee et al., 2010; Blamey et al., 2012; Lazard et al., 2012; Holden et al., 2013; Pisoni et al., 2016). However, the underlying mechanism for the large variability in CI outcomes is still not well understood. This lack of information is a barrier to customized rehabilitation and hampers further improvement of CI outcomes.

It is well known that the right and left hemispheres of the brain play distinct roles in processing auditory information. In normal hearing, the left hemisphere is dominant in processing temporal information and the right hemisphere is dominant for spectral information (Zatorre et al., 2002; Schonwiesner et al., 2005; Hyde et al., 2008; Okamoto et al., 2009). In adults wearing CIs, processing spectral information in CI users is substantially impaired due to the low number of spectral channels used to deliver sound information and the deafness-related neural deficits in the auditory system (Giraud et al., 2001; Limb and Roy, 2014). Thus, the side of cochlear implantation may influence the outcomes of the CIs, particularly for adult CI users. However, direct information for the effect of the implantation side on the CI outcome is not available.

The electroencephalography (EEG) technique allows for the recording of auditory evoked potentials with an excellent temporal resolution, enabling the examination of real-time brain processing. EEG is the most suitable tool to examine the neural substrates of sound processing in CI users (Debener et al., 2008). With EEG techniques, researchers have examined the cortically generated auditory evoked potential (CAEP), which consists of the N1 and P2 peaks occurring in a window of approximately 70–250 ms after stimulus onset. The acoustic change complex (ACC) is a special type of the CAEP elicited by an acoustic change (e.g., a change in frequency, intensity, duration, etc.) embedded in a stimulus (Ostroff et al., 1998; Abbas and Brown, 2014; Brown et al., 2015; Kim, 2015). Data from non-CI users showed that the ACC threshold (the minimum magnitude of the acoustic change required to evoke the ACC) is in agreement with behaviorally measured auditory discrimination thresholds and that the ACC amplitude is related to the salience of the perceived acoustic change (He et al., 2012; Liang et al., 2016; Fulbright et al., 2017). Moreover, multi-channel EEG data could be used in source localization analysis to identify the activated brain regions (Worrell et al., 2000; Plummer et al., 2010; Eugene et al., 2014; Talja et al., 2015).

Source localization techniques can be used to estimate the current source generators in the brain that best fit the scalp recorded EEG or MEG data. Although there is no unique solution to the neuroimaging inverse problems, the standardized Low-Resolution Brain Electromagnetic Tomography (sLORETA, available at http://www.uzh.ch/keyinst/loretaOldy.htm) can be used to calculate neural generators of EEG or MEG data with exact and zero error localization for test dipoles (Pascual-Marqui, 2002; Wagner et al., 2004; Grech et al., 2008). Moreover, sLORETA has no localization bias in the presence of measurement and biological noise (Pascual-Marqui, 2002). Comparing other techniques such as WMN and LORETA, sLORETA gives the best solution in terms of both localization error and sources (Grech et al., 2008) and this method has been validated both theoretically and experimentally (Pascual-Marqui, 2002; Plummer et al., 2010). Using sLORETA, it is possible to specifically compare the activity of regions of interest (ROIs) between the left and right hemispheres or to examine the correlations between brain activities and behavioral measures.

Previous ACC studies in CI users were restricted to the analysis of the ACC waveform without the identification of neural generators of the response (Friesen and Tremblay, 2006). Meanwhile, in the studies using source localization analysis in CI users, the analysis was limited to the response evoked by the onset of the stimulus (i.e., the onset CAEP, Wong and Gordon, 2009; Gordon et al., 2010; Song et al., 2013; Nash-Kille and Sharma, 2014) rather than the response evoked by acoustic changes embedded in the stimulus (i.e., ACC).

A study from our lab reported that the CI users exhibited much poorer ACC waveforms evoked by frequency changes compared to normal hearing subjects (Liang et al., 2018). Behavioral studies from other researchers using pitch discrimination tasks or spectral modulation tasks have also reported that the capability of detecting changes in the frequency domain is crucial for speech performance in CI ears (Gifford et al., 2014; Won et al., 2014; Kenway et al., 2015). Therefore, it is important to examine brain activation patterns to frequency changes in CI users to understand the neural basis of the CI outcomes. In the current study, we performed sLORETA source analysis using ACC data collected for Liang et al.’s (2018), in which only ACC waveform results were reported, to examine brain activation patterns in response to tones containing frequency changes in the adult CI users. The focus of the current study, which is a companion paper to Liang et al. (2018), is to examine the effect of the implantation side (right- and left-ear CIs) on the cortical processing of frequency changes. Therefore, the brain activation patterns of the right- vs. left-ear CIs and additional waveform comparisons will be presented. To our knowledge, the current study is the first one to investigate the effects of the implantation side on cortical processing of frequency changes and to localize the neural substrates of the ACC N1’ in CI users. This study will be valuable for guiding CI side selection and maximizing CI outcomes. We hypothesize that the brain activation patterns of the ACC N1’ peak evoked by frequency changes are correlated to the behaviorally measured frequency change detection threshold, which has been reported to significantly affect CI speech performance (Zhang et al., 2019). We also hypothesize that the brain activation patterns are different in right- and left-ear CIs in patients who are right-handed.

## Experimental Section

### Participants

Twelve CI users (seven females, five males; 43–75 years old, with a mean age of 63 years) wearing the devices from Cochlear (Sydney, Australia) participated in this study. All participants were right-handed and native English speakers with no history of neurological or psychological disorders. They did not take medications that have been reported to affect the EEG. The CI users had severe-to-profound, bilateral, sensorineural hearing loss prior to implantation. Of the twelve CI subjects, seven subjects were bilateral CI users, four subjects were bimodal device users (one ear wore a CI and the non-implanted ear wore a hearing aid), and one subject was a unilateral CI user. Each CI ear was tested individually. In one bilateral CI user, only one CI ear was recorded, because the other CI ear was not able to detect the maximum magnitude of frequency changes in the psychoacoustic test. Therefore, both EEG and behavioral data were collected from a total of 18 ears (ten right-ear CIs and eight left-ear CIs). Individual CI subject’s demographic information has been provided in Table 1 of Liang et al. (2018).

In addition, data from twelve normal hearing (NH) individuals (six females, six males; 20–30 years old, with the mean age of 23 years) were used to provide information on brain activity in individuals with a normal auditory system. The ACC waveform data from NH listeners have been reported in a previous study from our lab (Liang et al., 2016). The purpose of presenting the sLORETA data from NH listeners is not for direct comparison between the NH and CI group (since the stimuli were presented binaurally to the NH listeners and monaurally to each CI ear, Liang et al., 2016, 2018). Rather, the NH data presented here serves as a validation of the sLORETA methodology because the NH results could be compared to those in the literature. Moreover, discussion of the CI results, with the knowledge of NH results, would be beneficial for the understanding of the nature and degree of abnormalities in cortical processing of frequency changes in CI users. All NH listeners had audiometric hearing thresholds ≤20 dB HL at octave test frequencies from 250 to 8000 Hz. All participants gave informed written consent prior to their participation. This study was approved by the Institutional Review Board of the University of Cincinnati.

### Stimuli

The stimuli were 160 Hz tones generated using Audacity^1^ at a sample rate of 44.1 kHz. The duration of the tones was 1 s, including linear ramps of 10 ms at the onset and offset. The 160 Hz tone was selected because this frequency is in the range of the fundamental frequency (F_0_) of the human voice (between the F_0_ of female and male voiced speech, Gelfer and Bennett, 2013). The 160 Hz tone contained upward frequency changes of different magnitudes at 500 ms after the tone onset. The frequency change occurred for an integer number of cycles of the base frequency at 0 phase (i.e., zero crossing). Therefore, the onset cue of the frequency change was removed, and it did not produce audible transients (Dimitrijevic et al., 2008; Pratt et al., 2009). The stimuli were initially presented at 85 dB (peSPL) through a loudspeaker placed at ear level, 50 cm in front of the participant. CI users were tested using their typical everyday speech processor settings, but were allowed to adjust the volume so that the loudness level of the stimuli corresponded to the loudness level 7 (the most comfortable level) on a 0-10-point (inaudible to uncomfortably loud) numerical scale (Hoppe et al., 2001). The most comfortable level has been widely used in EEG studies involving CI users to minimize the loudness differences across CI users (Ponton et al., 1996; Friesen and Tremblay, 2006).” According to Pfingst et al. (1994), the mean frequency discrimination performance for CI users is improved when the loudness level increases up to level 4 and then does not change significantly up to loudness level 10.

### Psychoacoustic Test of the Frequency Change Detection Threshold

Participants were seated in a sound-treated booth for the testing. An adaptive, 2-alternative forced-choice procedure with an up-down stepping rule using APEX software (Francart et al., 2008) was employed to measure the frequency change detection threshold (FCDT). In each trial, a standard stimulus (the 160 Hz tone) and a target stimulus (the 160 Hz tone containing a frequency change with a magnitude of up to 65%; the step size was 5% from 10–65% range, 0.5% from 0.5–10% range, and 0.05% from 0.05–0.5% range) were included. The order of standard and target stimulus was randomized and the interval between the stimuli in a trial was 0.5 s. The participant was instructed to choose the target signal by pressing a button on a computer screen and was given a visual feedback regarding the response. Each run generated a total of five reversals. The asymptotic amount of frequency change (the average of the last three trials) was used as the FCDT. Each CI ear was tested separately. When one CI ear was tested for bilateral users, the opposite CI was turned off. Bimodal users were only tested on the CI side with the hearing aid on the contralateral side turned off and blocked with an earplug.

### EEG Recording

Participants were seated on a comfortable chair in a sound-treated booth for the experiments. A 40-channel Neuroscan multi-channel EEG system (NuAmps, Compumedics Neuroscan, Inc., Charlotte, NC) was used to record the EEG. Electro-ocular activity (EOG) was monitored so that eye movement artifacts could be identified and rejected during the offline analysis. The continuous EEG data were recorded with a band-pass filter setting from 0.1 to 100 Hz and a sampling rate of 1,000 Hz. The average electrode impedance was lower than 10 kΩ. EEG signals from a total of 1–3 electrodes over the CI coil were not available.

During testing, participants were instructed to avoid excessive eye and body movements. Participants read self-selected magazines to keep alert and were asked to ignore the acoustic stimuli. A total of 400 trials of each of the three types of stimuli (three frequency changes: 0%, 5%, and 50%) were presented. The stimulus conditions were randomized to prevent order effects. The inter-stimulus interval was 800 ms. Each CI ear was tested separately.

### Data Processing and Statistical Analysis

#### Waveform Analysis

The detailed procedures for waveform analysis were described in Liang et al. (2018). Briefly, continuous EEG data collected from each participant were digitally filtered (0.1–30 Hz) and then divided into segments over windows of -100 ms to 1000 ms relative to the tone onset. Each data segment was visually inspected, and epochs contaminated by non-stereotyped artifacts were rejected and excluded from the analysis (Delorme and Makeig, 2004). There were at least 200 epochs left for each participant. Further data processing was performed by the use of the EEGLAB toolbox (Delorme and Makeig, 2004) running under MATLAB (MathWorks, United States). The data were baseline-corrected and then re-referenced using a common average reference. Independent component analysis (ICA) was then applied to identify and remove non-neurological activities (Gilley et al., 2006; Debener et al., 2008; Zhang et al., 2009, 2011). EEG from the electrodes close to the CI coil were replaced by linearly interpolated values computed from neighboring EEG signals. Then, the averaged waveform was derived for each type of stimuli (0%, 5%, and 50% frequency change) separately. The N1’peak of the ACC was identified in the range of 610–710 ms after stimulus onset or 110–210 ms after the occurrence of the frequency change.

Only EEG data elicited by the 50% frequency change were used for source density reconstruction because the ACC was present in all CI ears for the 50% frequency change, but was missing in some CI ears for the 5% frequency change and absent for the 0% change (see Liang et al., 2018). The presence of the ACC was determined based on criteria: (i) an expected wave morphology within the expected time window (approximately 610–710 ms after the tone onset) based on mutual agreement between two researchers (Lister et al., 2011; He et al., 2015); and (ii) a visual difference in the waveforms between the frequency change conditions vs. no change condition.

#### Sloreta Analysis

The waveform data were further imported into the sLORETA software package for source localization for the negative peak evoked by the frequency change (ACC N1’). The data from the right-ear CIs and left-ear CIs were analyzed separately. The following processes were conducted for sLORETA analysis: (1) The 3-dimensional sLORETA maps were generated to show the current source density (CSD) distribution patterns of ACC N1’ (a timeframe ranging from 610 ms to 710 ms after stimulus onset); and (2) Regions of Interests (ROIs) were defined based on the activated brain regions observed in individual CIs. The correlations between the activities in the ROIs and the FCDT were also examined. The following ROIs of four brain regions were examined: the left and right temporal lobe (including Brodmann areas 21, 22, 38, 39, 41, and 42), and the left and right frontal lobe (including Brodmann areas 6, 9, 10, 11, 44, 45, 46, and 47) (Campbell and Sharma, 2013). These ROIs were selected because the greatest activities are in the temporal lobes and frontal lobes in the mean current source density distribution patterns of the ACC N1’. The ROI file for the 4 seed points for the center voxel was constructed. Each of the ROI values consisted of the mean current source density from each ROI seed, including all cortical gray matter voxels within a 15-mm distance from the center. The resulting file produced the log transformed average CSD for all participants for each seed (Cannon and Baldwin, 2012). The CSD data for each brain region were imported into the Sigmaplot software program for statistical analyses.

#### Statistical Analysis

Data were presented as mean ± SEM and plotted with SigmaPlot v10 (SPSS Inc., Chicago, IL, United States). The statistical analyses were performed by Excel (Microsoft Office 365) or SPSS (SPSS Inc.) using *t* test or AVOVA with a Bonferroni correction for the *post hoc* tests. The level of statistical significance was set at *p* < 0.05. All sample sizes and P values were reported in the figures or the figure legends.

## Results

### Event-Related Potentials and ACC in Right-ear CIs, Left-ear CIs, and NH Subjects

**FIGURE 1:**
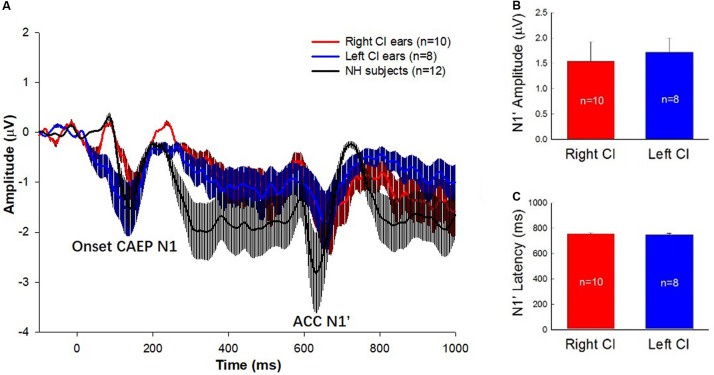
**(A)** ERP waveforms with error bars for the right-ear CIs (red traces), left-ear CIs (blue traces), and the NH listeners (black traces) are displayed. The stimulus was a 1-s pure tone at 160 Hz that contained an upward frequency change of 50% in the middle of the stimulus. The N1 peak of the onset CAEP evoked by the tone onset and the N1’ of the ACC evoked by the 50% frequency change are marked. Note that the N1’ is the focus of this study. **(B,C)** The means and standard errors of the N1’ amplitude and latency for left- and right-ear CIs. There is no significant difference in the amplitude and latency of N1’ waves between right- and left-ear CIs.

Figure 1A shows the mean event-related potentials (ERPs, solid lines) and the standard errors at Cz evoked by the 160 Hz tones containing a 50% change for the right-ear CIs (red trace), left-ear CIs (blue trace), and NH listeners (black trace). As shown by the figure, there were two types of responses visible in the waveforms. One is an onset CAEP with the N1-P2 complex occurring approximately 110–210 ms after the onset of stimuli. The other one is the ACC with the N1’-P2’ complex occurring approximately 610–710 ms after stimulus onset or 110–210 ms after the occurrence of the frequency change. There is a prominent difference in the amplitude and latency of ACC N1’ between NH and CI subjects. Figures 1B,C show the means of N1’ amplitude and latency for left- and right-ear CIs. The t-tests showed that there were no significant differences in N1’ amplitude and latency between right- and left-ear CIs (*t* = 0.16, *df* = 16, *p* = 0.87 for latency; *t* = -0.35, *df* = 16, *p* = 0.73 for amplitude).

### Difference in Activated Brain Regions in Right-ear CIs, Left-ear CIs, and NH Subjects

**FIGURE 2:**
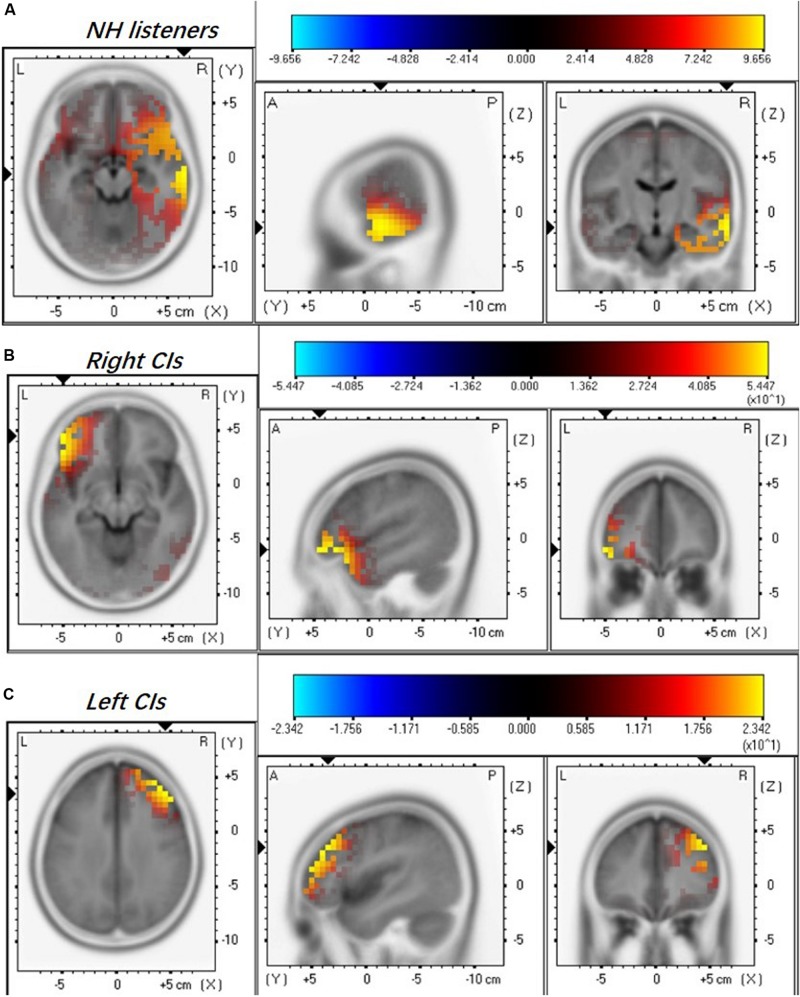
Mean regional normalized sLORETA solutions modeling the distributed sources for ACC N1’ of NH listeners **(A)**, right-ear CIs **(B)**, and left-ear CIs **(C)**. Yellow and blue colors represent increased and decreased current source density, respectively. Note that the NH listeners were stimulated binaurally and each CI ear was stimulated monaurally. There are differences in the activated brain regions among NH, right-, and left-ear CIs.

Current source density (CSD) maps derived using the sLORETA reflect the intensity of activation in the brain regions evoked by the presented stimulus. Figure 2 illustrates the CSD patterns of the ACC N1’ peak in the grand mean waveform for NH listeners, right-ear CIs, and left-ear CIs. In NH listeners, the brain activation is mainly visible in the right temporal lobe (Figure 2A). However, the CI ears show an apparent difference in the activated brain regions (Figures 2B,C). For the right-ear CIs, the increase in CSD is visible in both the left temporal lobe and left frontal lobe (Figure 2B), while for the left-ear CIs the CSD increase is only visible in the right frontal lobe (Figure 2C).

### Good Performers in Frequency Change Detection Show More Activation in the Temporal Lobe

**FIGURE 3:**
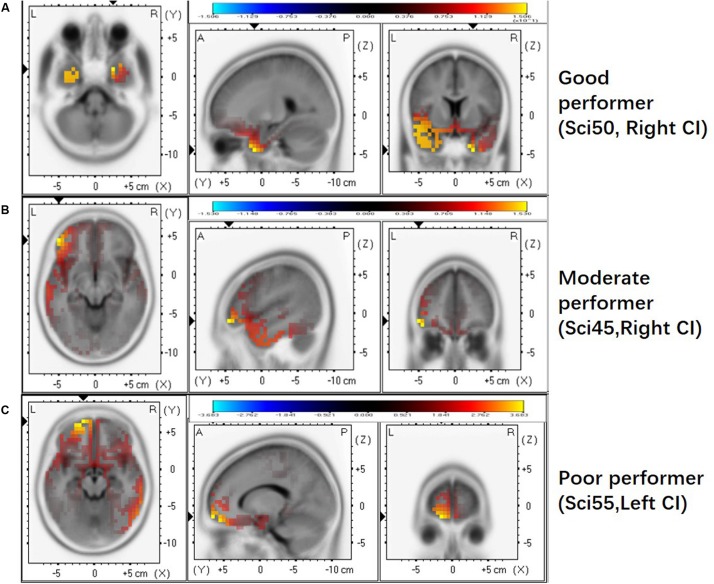
The activated brain regions displayed for individual CI users with different FCDTs. **(A)** good performer (SCI 50 R, 1.83%). **(B)** poorer performer (SCI45 R, 5.17%). **(C)** poorest performer (SCI55 L, 9.33%). The strong activation in the temporal lobe appears in the good performer with a low FCDT.

We further examined whether the activated brain regions are associated with performance on the frequency change detection task. Figure 3 shows brain activation patterns for ACC N1’ peak in a good performer (Sci50R, FCDT: 1.83%), a moderate performer (Sci45R, FCDT: 5.17%), and a poor performer (Sci55L, FCDT: 9.33%) in the frequency change detection task. The good performer with low FCDTs showed more activation in the temporal lobe compared to the frontal lobe (Figure 3A), whereas the moderate and poor performers with high FCDTs had more activation in the non-temporal lobe including the frontal lobe (Figures 3B,C). The good or poor performance on the frequency change detection task was defined by comparing the FCDTs across all subjects tested. Our earlier publication using the FCDT task to examine the ability to detect frequency changes in CI users at base frequencies of 250, 1000, and 4000 Hz reported that poor performers (CNC, AzBio quiet and noise, and triple digit test) showed FCDTs of approximately 10% and above, while good performers showed a FCDT of approximately 1%. Using other methods (pitch discrimination/ranking), other studies also examined frequency discrimination in CI users. For example, Goldsworthy (2015) reported that CI users’ pitch discrimination thresholds for pure tones (0.5, 1, and 2 kHz) ranged between 1.5 and 9.9%.

### Difference in the Hemisphere Dominance Patterns of Current Source Density in Right- and Left-ear CIs

**FIGURE 4:**
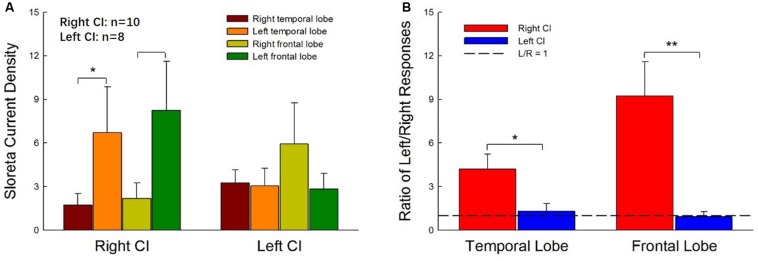
Hemispheric differences in the current source density values for right- and left-ear CIs. **(A)** The current source density values in four regions of interest (ROIs, right, and left temporal lobes and frontal lobes) for right- and left-ear CIs. For the right-ear CIs, the current source densities in the contralateral hemisphere (left temporal lobe and left frontal lobe) are larger than those in the ipsilateral (right) hemisphere. The contralateral-dominant pattern is not observed in the left-ear CIs. **(B)** The left-dominant pattern is obvious in the right-ear CIs but not visible in the left-ear CIs. The left/right (L/R) ratios of responses in both the temporal lobe and frontal lobe for the right-ear CIs are greater than 1, whereas the L/R ratios of responses in both the temporal lobe and the frontal lobe for the left-ear CIs are close to 1. There are significant differences in the ratio of L/R responses between right-ear CIs and left-ear CIs (*p < 0.05, **p < 0.01, *t*-test).

Figure 4 shows the mean CSD values in the four ROIs, the right and left temporal and frontal lobes, for right- and left-ear CIs. In general, stimulation in the right-ear CIs resulted in larger CSD values in the contralateral (left) hemisphere (Figure 4A) than in the ipsilateral (right) hemisphere. For the left-ear CIs, the activation in the contralateral (right) frontal lobe appeared to be stronger than that in the ipsilateral (left) side and the hemispheric difference did not exist in the temporal lobes. The t-tests showed that the activation is significantly stronger in the temporal and frontal lobes of the left side than that of the right side for the right-ear CIs (Figure 4A, *t* = -2.23, *df* = 18, *p* = 0.04 and *t* = -2.31, *df* = 18, *p* = 0.03, respectively, for the temporal and frontal lobe comparisons). One-way ANOVA did not show significant difference among the four ROIs for the left-ear CIs [*F*_(__1_,_3__)_ = 1.54, *p* = 0.31]. The hemispheric difference was further examined with the response ratio of the left/right (L/R ratio) hemispheres and the results were shown in Figure 4B. For the right-ear CIs, the L/R ratios in the temporal lobe and the frontal lobe were 4.20 ± 1.30 and 9.24 ± 2.35, respectively. For the left-ear CIs, the L/R ratios of responses in the temporal lobe and the frontal lobe were 1.30 ± 0.519 and 0.93 ± 0.34, respectively (Figure 4B), indicating that there was no apparent difference in the responses evoked in the left and right brain regions for the left-ear CIs. When comparing the right- and left-ear CIs, the L/R ratios of responses in both temporal lobe and frontal lobe for the right-ear CIs were significantly higher than these for the left-ear CIs (Figure 4B, *t* = 2.50, *df* = 16, *p* = 0.026 and *t* = 3.13, *df* = 16, *p* = 0.006 for temporal and frontal lobes, respectively). In summary, the contralateral dominance of brain activation was observed for the right-ear CIs, but not for the left-ear CIs.

### Correlation Between Activities in Temporal and Frontal Lobes and FCDT

**FIGURE 5:**
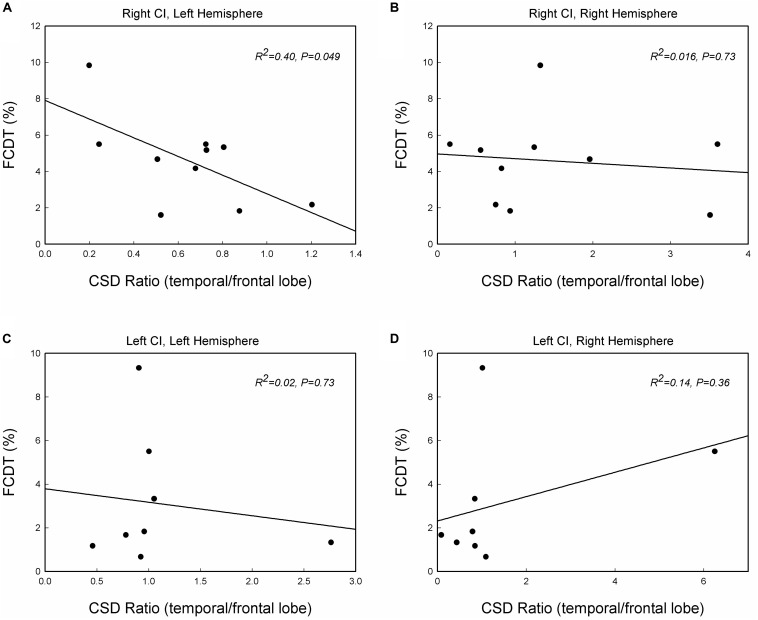
The correlations between the FCDT and the ratio of current source density (CSD) values in the temporal lobe and frontal lobe in the left and right hemispheres for the right-ear CIs **(A,B)** and left-ear CIs **(C,D)**. Straight lines represent linear regression lines. Note that only the right-ear CIs in panel **(A)** shows a significant correlation, with good performers (low FCDTs) showing larger CSD ratios in the left hemisphere (*p* < 0.05).

Previous studies suggested that the auditory change detection depends on the interaction between the temporal and the frontal cortex (Doeller et al., 2003). We further examined the correlation between FCDT and the interactive activations in the temporal lobe and frontal lobe. Figure 5 shows the scatter-plot of the FCDT vs. the CSD ratio (temporal lobe/frontal lobe) in the right and left hemispheres for right-ear and left-ear CIs. The CSD ratio in the left hemisphere for the right-ear CIs was negatively correlated to the FCDT (*R^2^* = 0.40, *p* = 0.049, Figure 5A). However, there was no significant correlation between FCDT and CSD ratio in the right hemisphere for the right-ear CIs. In addition, no statistically significant correlation was found for the FCDTs and the CSD-ratio for the left-ear CIs (*p* > 0.05. Figures 5C,D).

## Discussion

In this study, we examined the brain responses of right-ear CIs and left-ear CIs in right-handed adults. The primary findings were: (1) cortical activation patterns for the ACC-N1’ are different in right-ear CIs and left-ear CIs (Figures 1, 2); (2) Right-ear CIs could evoke stronger activities in the contralateral temporal and frontal lobes, but this contralateral hemisphere dominance was not observed for left-ear CIs (Figures 3, 4), and (3) For the right-ear CIs, the increased activation in the left temporal lobe, along with the reduced activation in the frontal lobe (increased temporal/frontal CSD ratio), is correlated with good performance in the detection of frequency changes (Figure 5). Such a correlation was not found for the left-ear CIs. These results suggest that in adults, the implantation side can significantly influence the brain activation patterns evoked by the within-stimulus frequency changes.

Previous studies on brain activation patterns in CI users have focused on the onset CAEP, i.e., the cortical auditory evoked response to stimulus onset (Debener et al., 2008; Sandmann et al., 2009). Using dipole source analysis, Debener et al. (2008) reported the onset CAEP in one patient who had successfully used a CI in the right ear for four years. The results showed that the contralateral response is larger than the ipsilateral response. Sandmann et al. (2009) examined the onset CAEP to dyadic tones with pitch intervals that sound like music in CI users and NH listeners. While NH listeners showed a contralateral dominance effect for the left ear stimulation (Hine and Debener, 2007), CI users showed a contralateral dominance effect in the auditory regional source activity specifically for the right-ear stimulation. This suggested that the hemispheric asymmetry in CI users differs from that in NH listeners.

Brain activation patterns for the ACC N1’ peak has not been studied in CI users. Unlike the onset-CAEP that indicates the cortical processing of stimulus onset, the ACC indicates the cortical processing of acoustic change embedded in a stimulus. In NH listeners, the processing of frequency changes is specialized in the right hemisphere, with either a binaural or monaural stimulation (Liegeois-Chauvel et al., 2001; Zatorre and Belin, 2001; Zatorre et al., 2002; Molholm et al., 2005; Schonwiesner et al., 2005; Dimitrijevic et al., 2008; Hyde et al., 2008; Itoh et al., 2012). When the acoustic change is small, the frontal lobe may also be activated (Opitz et al., 2002; Schönwiesner et al., 2007). Lesion studies also suggest that damage in the right hemisphere results in an impaired capability to discriminate frequencies, supporting that the right hemisphere has a specialized function of processing spectral changes (Sidtis and Volpe, 1988; Zatorre, 1988; Robin et al., 1990; Johnsrude et al., 2000). The activation in the right temporal lobe for the NH listeners’ ACC N1’ in the current study (Figure 2) is consistent with what has been reported in previous studies.

This study provides evidence that the temporal lobe and frontal lobe are involved in frequency change detection in CI users. This study also shows that CI users demonstrated brain activation patterns for the ACC N1’ that are different from those in NH listeners. While the right-ear CIs result in activation in the contralateral temporal and frontal lobes, the left-ear CIs generate less activation in the contralateral temporal lobe (Figures 3, 4). The differences in the brain activation patterns in the CI users relative to the NH listeners may arise from CI technology limitations and/or brain reorganization in CI users. Specifically, the CI delivers the sound information through a limited number of frequency channels, resulting in a dramatic decrease in CI users’ frequency resolution. Such a compromised frequency resolution is exacerbated by the neural deficits related to the long-term deafness prior to the implantation. In this study, 160 Hz was used because this frequency is in the frequency range of the fundamental frequency (F0). As this frequency is located at the filter slope of the first band at the CI speech processing stage, the detection of a 50% frequency change (from 160 Hz to 240 Hz) may rely on the temporal rate cues as the result of different signal intensities on the filter response curve in the CI output, and/or the place cues at a result of activating different electrodes. Therefore, we speculate that frequency change detection relies on both temporal and spectral cues in CI users. The ACC results from CI users in the current study suggested that the auditory brain can automatically encode the 50% frequency change from the 160 Hz, although the activation pattern may differ from that in NH listeners.

We found that the CI-activated brain regions included the temporal lobe as well as non-temporal regions, such as the frontal lobe (Figures 2–4). Moreover, the brain activation and activated patterns were related to the behaviorally measured FCDT (Figures 3, 5). Specifically, the right-ear CIs with good performance had similar brain activation patterns as the NH listeners, mainly activating the temporal lobe (Figures 3, 4). The CI ears with poor performance were likely to have activation in non-temporal areas (Figure 3). The ears with better performance had a greater temporal lobe/frontal lobe CSD ratio (stronger activation in the temporal lobe and weaker activation in the frontal lobe) on the contralateral side for the right-ear CIs (Figure 5A). Green et al. (2005) reported that the activation strength in the temporal lobe is positively correlated with the speech perception performance (Green et al., 2005). In future studies, it is necessary to evaluate the correlations between the ACC brain activation and speech perception performance in CI users.

The major finding in this study is that in comparison with left-ear CIs, right-ear CIs can more efficiently activate the temporal lobe in the contralateral hemisphere (Figures 3, 4). Previous studies examining the onset cortical responses have reported that the contralateral dominance is greater in CI users using a right CI compared to a left CI (Debener et al., 2008; Sandmann et al., 2009). Studies using neuroimaging techniques, such as positron emission tomography (PET), also showed a higher activation in the cortex contralateral to the CI than that on the ipsilateral side (Herzog et al., 1991). In this study, we not only used sLORETA to locate activated brain regions but also examined the behavioral measure of frequency change detection. We found that, in comparison with left-ear CIs, the right-ear CIs are more efficient in activating the contralateral brain regions (Figures 3–5).

It is unknown why the right-ear CIs in this study demonstrated more activity in the contralateral hemisphere than in the ipsilateral hemisphere, while the left-ear CI s did not show this contralateral dominance. One possibility is that such a phenomenon is related to the fact that all tested subjects in this study were right-handed. Brain activations evoked by acoustic stimuli show different patterns for right-handed and left-handed NH individuals (Provins and Jeeves, 1975; Maehara et al., 1999). It is possible that the pre-existing hemispheric differences for right-handed individuals before implantation may be differentially affected by right- vs. left-ear implantation. A review study (Kraaijenga et al., 2018) based on 20 articles that were eligible for critical evaluation of the effect of the implantation side on CI outcomes reported that the majority of studies reveal evidence for a right-ear advantage in post-lingually deafened adults. The right-ear advantage was also reported in prelingually deafened children wearing CIs (Henkin et al., 2014). Although it is too premature to advise implanting in the right ear, our findings in the current study do support the idea that cortical processing of frequency changes show different brain activation patterns between the right- and left-ear CIs, at least in right-handed adults.

## Conclusion and Future Studies

This study reveals that the side of implantation can significantly influence the brain activation patterns evoked by frequency changes in adults with right-handedness. Right-ear CIs result in stronger brain activities in the contralateral hemisphere than in the ipsilateral hemisphere. Such a contralateral dominance was not observed for left-ear CIs. For right-ear CIs, good performance in frequency change detection is correlated with larger temporal activation, along with weaker frontal activation. The findings of this study provide valuable evidence that the right-ear implantation appears to support the contralateral dominance for right-handed patients. The data also demonstrated that the sLORETA is a promising source location approach and can be used to longitudinally examine brain plasticity after cochlear implantation, to examine the development of auditory cortex reorganization, to actively guide rehabilitation strategies, and to monitor the progress of an individual during rehabilitation.

There are some possible directions for future studies. First, we will further examine the brain activation patterns to frequency changes in both left-handed and right-handed CI patients with a larger sample size. With a larger sample size, the correlation observed in Figure 5 of this study might be stronger; second, it would be valuable to add NH listeners who are age-, gender-matched, and test to their individual ears corresponding to those in their CI counterparts. Finally, the CI outcomes in terms of speech perception will be obtained to establish the brain-behavioral relationships.

## Data Availability Statement

The datasets generated for this study are available on request to the corresponding authors.

## Ethics Statement

This study involving human participants was reviewed and approved by Fawen Zhang’s affiliated institution, University of Cincinnati. The patients/participants provided their written informed consents to participate in this study.

## Author Contributions

CL and FZ conceptualized, formally collected and analyzed the data. CL, FZ, RS, JX, and LW contributed to patient recruitment and methodology. CL and FZ prepared and wrote the manuscript. All authors reviewed and edited the manuscript.

## Conflict of Interest

RS and LW received research and travel funding from Cochlear Ltd. The funders had no role in the design of the study, the collection, analyses, or interpretation of data, the writing of the manuscript, or the decision to publish the results. The remaining authors declare that the research was conducted in the absence of any commercial or financial relationships that could be construed as a potential conflict of interest.

## Footnotes

1 http://audacity.sourceforge.net
